# Survey of German veterinarians' approaches to pain assessment and management of perioperative pain in pet rabbits

**DOI:** 10.1002/vetr.6018

**Published:** 2025-12-04

**Authors:** Stephanie Zein, Katharina C. Jensen, Kerstin Müller

**Affiliations:** ^1^ Division for Small Mammals, Birds and Reptiles Small Animal Clinic School of Veterinary Medicine Freie Universität Berlin Berlin Germany; ^2^ Institute for Veterinary Epidemiology and Biostatistics School of Veterinary Medicine Freie Universität Berlin Berlin Germany

## Abstract

**Background:**

This study aimed to assess veterinary practices in pain recognition and perioperative analgesic therapy in pet rabbits.

**Methods:**

An online questionnaire was distributed German veterinarians from July to October 2022, containing questions on the frequency with which they treated rabbits and the methods they used to evaluate pain in this species. Additionally, participants were asked detailed questions about their approach to perioperative pain management when performing soft‐tissue surgery, specifically an ovariohysterectomy, in rabbits.

**Results:**

One hundred and fifty‐four questionnaires were considered for final analysis. The most commonly reported indicators to detect pain in rabbits were assessment of food intake and behavioural observation. A total of 23.4% of the participants stated that they used the Rabbit Grimace Scale. Overall, 24.5% of the 110 veterinarians who performed ovariohysterectomies in rabbits reported no use of preoperative analgesia; however, 95.5% administered multimodal analgesia in the pre‐ and intraoperative phases combined and 60.0% administered analgesia postoperatively. The most commonly mentioned drug combination pre‐ and postoperatively was metamizole and meloxicam. Opioids and local anaesthetics were used less frequently.

**Limitations:**

The survey had a small sample size and notable selection bias, with 90.3% female participants.

**Conclusion:**

Respondents used different indicators to detect pain in rabbits. Analgesic therapy in ovariohysterectomies, as reported by participants, can be considered suboptimal in several cases.

## INTRODUCTION

Rabbits are the third most common pet in Germany after dogs and cats,^1^ and they are presented to veterinary practices for routine examinations, minor and elective procedures, and major medical interventions. Recognising pain and treating it appropriately is an essential part of all patient management, but veterinarians face several challenges with rabbits in this regard.

First, veterinarians must be able to interpret clinical and behavioural signs of pain in rabbits, which are often subtle and inconspicuous, as rabbits are typical prey animals.^2^, ^3^ Several studies in recent years have expanded the possibilities of pain recognition in rabbits, starting with the development of the Rabbit Grimace Scale (RbtGS)^4^ and leading to the development of complex pain scales such as the Bristol Rabbit Pain Scale (BRPS), the Rabbit Pain Behaviour Scale (RPBS) and Centro Animali Non Convenzionali Rabbit Scale (CANCRS).^5^, ^6^, ^7^ Pain assessment in rabbits has thus become more sophisticated. However, the tools have not yet been sufficiently tested for their clinical applicability and practicality for practitioners.

Second, there is a significant lack of clinical trials on analgesic therapy, particularly multimodal analgesic therapy, in rabbits.^3^ Almost no analgesic drugs are licensed for the use in rabbits in the European Union and the dosages of several analgesics for rabbits, summarised from a few studies and otherwise from textbooks, vary considerably.^8^ This raises the question of which methods veterinarians use and how confidently they recognise and treat pain in pet rabbits in their daily work.

While there are multiple studies on the assessment and management of pain by veterinary surgeons in various countries concerning dogs and cats,^9^, ^10^, ^11^, ^12^, ^13^, ^14^ as well as larger animals such as cattle^15^, ^16^, ^17^, ^18^, ^19^, ^20^, ^21^, ^22^, ^23^ and horses,^16^, ^21^ only one study has been carried out in New Zealand^24^ and one in the UK^25^ regarding pet rabbits.

Therefore, this questionnaire‐based study aimed to investigate the attitudes of veterinarians in Germany towards pain assessment and management in pet rabbits. Specifically, perioperative analgesic therapy was to be assessed by asking participants to provide detailed information about their analgesic protocol for performing an ovariohysterectomy (OHE) in rabbits, since it is a standardised routine soft‐tissue surgery comparable to other species, which is performed in many female pet rabbits and not only carried out by specialists.^26^

## MATERIALS AND METHODS

### Questionnaire

An online survey in German with a total of 24 questions was created via LimeSurvey, categorised into three parts, beginning with general questions on pain assessment and pain therapy in rabbits, followed by specific questions on perioperative analgesic therapy for OHEs in rabbits, and finally, questions on the demographics of the participants. After developing the preliminary questionnaire, it was tested by six colleagues (veterinarians and veterinary nurses) as well as the authors themselves. The questionnaire was then further revised to improve its feasibility and clarity. The full questionnaire in English is provided in the Supporting Information.

The questions of the final survey were specifically pertaining to the following:

- The frequency with which participants treated rabbits, indicators to assess pain in rabbits and the participants' confidence to recognise pain in the species, as well as the quality of participants' university education on the subject.
- Whether participants performed OHEs in rabbits and, if so, how frequently, followed by detailed questions on drugs, dosages and route of administration used in the pre‐, intra‐ and postoperative phases of an OHE (with the patient being a young animal of approximately 1 year of age, American Society of Anesthesiologists (ASA) classification group 1), postoperative management and time of discharge, follow‐up checks and the veterinarians' opinions on the quality of their chosen pain therapy.
- Demographic information on participants' gender, location of practice, year of graduation, time spent in practice and postgraduate specialisation in small mammal medicine. As only two veterinarians in a German‐speaking country held the European College of Zoological Medicine (ECZM) degree in small mammal medicine, this qualification was not specifically asked about in order to keep the survey anonymous.

Most of the questions in the survey were designed as multiple choice. In addition, participants had the opportunity to write comments. The response field for the indicators and methods used to assess pain in rabbits was created as a free‐text field. The participants were also required to manually enter the dosages of the analgesic drugs used perioperatively, specified in mg/kg.

The invitation to participate on the survey was publicised on veterinary online platforms and via several veterinary newsletters (kleintier konkret, Kleintierpraxis, Vetline Newsletter). Additionally, members of the small mammal working group of the German Veterinary Association (DGK‐DVG) were invited to participate via mail. The data were stored and analysed anonymously.

### Data analysis

The survey was conducted over the period of 4 months (July–October 2022). All questionnaires received before 1 November 2022 were considered for analysis. A total of 233 participants took part in the survey. As some of the survey's questions were not completed or several answers remained unfinished by participants, only 154 questionnaires were ultimately included in the analysis. Seventy‐five participants abandoned the questionnaire before completing it, mainly in the first quarter of the survey, which corresponds to a total dropout rate of 32.2%. The completion of the questionnaire required approximately 20 minutes.

The data were saved using Microsoft Excel (version 2312) and analysed using IBM SPSS Statistics (version 29.0.0). Results of descriptive statistical analysis are reported as percentages and refer to the total number of participants (*n* = 154), except for the specific questions on OHEs, which were answered by the 110 participants who stated that they actually carried out OHEs in rabbits. This is indicated accordingly in the results section. Participants' responses to indicators used for pain recognition in rabbits were subsequently categorised using inductive coding. Based on descriptive analyses, the data were checked for plausibility. Only single data points were excluded from analysis (e.g., one participant's statement that he had received his licence to practise in 1965). Although all participants provided information on which analgesic drugs they used, information on dosages, route of administration and dosing intervals could only be analysed to a limited extent, and answers needed post‐processing as these details were often entered incompletely or inconclusively. The data were further analysed using chi‐squared and Fisher's exact tests (both two sided). *p*‐Values of less than 0.05 were considered statistically significant.

## RESULTS

### Demographics

The demographic data of respondents are presented in Table 1. The vast majority of the participants were working in Germany (96.1%, 148/154). Most of the participants were female (90.3%, 139/154). The median year of participants obtaining their licence to practice was 2007 (range 1980‒2022). Respondents working in a clinic or practice without a veterinarian specialised in small mammal medicine represented more than two‐thirds of the total (70.1%, 108/154).

**TABLE 1:** Demographic data of respondents (*n* = 154) to the online survey on pain assessment and management in pet rabbits.

Demographics	%	*n*
**Country of work**		
Germany	96.1	148
Austria	2.6	4
Not specified	1.3	2
**Gender**		
Female	90.3	139
Male	7.8	12
No answer	1.9	3
**Year veterinary license obtained (categorised answers)**		
Before 1990	11.7	18
1990—2000	19.5	30
2001—2010	27.9	43
2011—2020	38.3	59
After 2020	2.6	4
**Postgraduate degree/specialisation (multiple answers possible)**		
Veterinary specialist in small mammal medicine^a^	9.7	15
Additional qualification in small mammal medicine^b^	10.4	16
Listed member of the DVG Small Mammals Working Group^c^	16.9	26
None of the above—but at least two courses of further education on small mammal medicine per year are completed	52.0	80
None of the above	24.0	37
**Participants working with a specialised veterinarian in small mammal medicine in‐house**		
Yes	29.9	46
No	70.1	108
**Frequency of treatment of rabbits**		
Daily	45.5	70
Several times a week	26.0	40
Weekly	16.2	25
1‒3× in a month	11.7	18
<1× a month	0.6	1

^a^ The title can only be obtained in some federal states in Germany but is recognised in all. A 4‐year period of training under the supervision of a designated specialist is usually required, next to a certain caseload and continuing education hours in small mammal medicine (referring to rabbits, rodents, ferrets and other exotic mammals), a scientific publication in the field and the passing of an oral examination.

^b^ An additional qualification in small mammal medicine in Germany can be achieved upon completion of usually 2 years of work experience under the supervision of a veterinarian with training authorisation. A certain caseload, case reports and training hours must be documented before an oral examination can be taken. Regulations may vary from state to state.

^c^ The small mammals working group of the German Veterinary Association (DVG AG Kleinsäuger) is a group of veterinarians who are particularly interested in the welfare and medicine of small mammals. The list of designated veterinarians of the working group should help pet owners to find a veterinarian who has proven expertise in the field.

Nearly half of respondents stated that they treated rabbits on a daily basis (45.5%, 70/154), and an additional 26.0% (40/154) stated that they did so at least several times a week.

### Pain assessment in rabbits

A total of 61.7% (95/154) felt ‘fairly confident’ and 22.1% (34/154) felt ‘very confident’ in identifying pain in rabbits. Only 15.6% (24/154) in total reported that they felt ‘insecure’ or ‘rather insecure’ on the subject. The response of one participant was inconclusive. The participants who treated rabbits more frequently were more confident in recognising pain in rabbits (*p* < 0.001), whereas respondents’ year of graduation had no significant impact on their confidence in pain assessment (*p* = 0.092).

**FIGURE 1:**
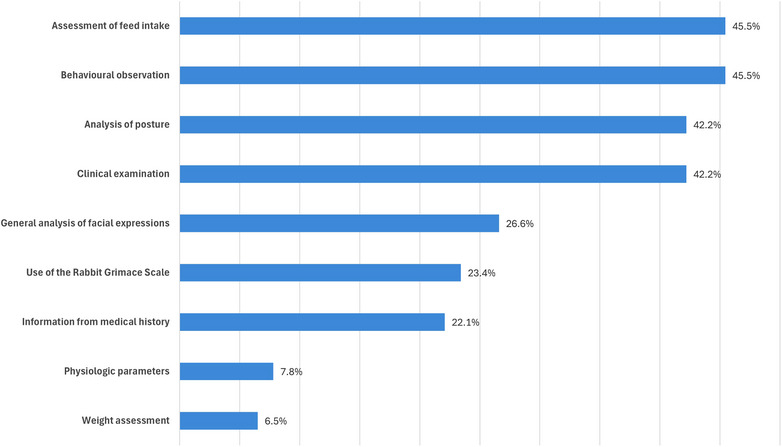
Percentage of indicators and methods used to recognise pain in rabbits as reported by survey participants (*n* = 154; categorised free‐text responses, multiple answers possible).

The vast majority of participants provided multiple reference points or methods for identifying pain in rabbits (95.5%, 147/154). A total of 45.5% of each (70/154) assessment of food intake and observation of behaviour were mentioned most frequently, followed by analysis of posture and the findings of the clinical examination, with 42.2% each (65/154) (Figure 1). A total of 23.4% (36/154) of respondents stated that they used the RbtGS for pain identification, and a further 26.6% (41/154) reported that they analysed the animals' facial expressions to determine pain but did not specifically use the term ‘(Rabbit) Grimace Scale’. A total of 22.1% (34/154) stated that they combined an analysis of posture with facial expressions or the RbtGS. A total of 5.2% (8/154) reported using a combination of analysis of posture, behavioural assessment and the RbtGS. Likewise, another 5.2% (8/154) reported combining physiological parameters or the findings of the clinical examination with facial expressions or the RbtGS. The participants that graduated after 2010 used the RbtGS more often than those who graduated earlier (*p* < 0.001). Other pain scales for rabbits were not specifically mentioned by participants.

With 87.7% (135/154), the vast majority felt that their veterinary university education had prepared them inadequately or not at all for the treatment of pain in rabbits and small mammals. A total of 45.5% (70/154) stated that they had been prepared ‘inadequately’, 42.2% (65/154) ‘not at all’ and 9.1% (14/154) ‘sufficiently’. A total of 1.9% (3/154) stated that they had been prepared ‘well’. No participant answered this question by selecting ‘very well’. The answers of two respondents were inconclusive. The response to evaluation of academic education was not associated with year of graduation, even when categorised by decade (*p* = 0.132).

### Perioperative pain management in rabbits undergoing OHE

#### Preoperative analgesia

More than two‐thirds (71.4%, 110/154) of respondents carried out OHEs in rabbits, about half of them less than once a month (52.7%, 58/110), 27.3% (30/110) one to three times a month and 20.0% (22/110) on a weekly basis or more often. Most rabbits were brought in on the day of the planned OHE (88.2%, 97/110).

Although there are no licensed analgesics for rabbits in Germany and the European Union, with the exception of a few ketamine products, the participants reported working with a variety of medications. An overview of all analgesics used perioperatively, with provided details on dosages and routes of administration, is presented in Tables 2, 3, 4.

**TABLE 2:** Preoperative use of analgesics in ovariohysterectomy of rabbits as stated by survey participants (*n* = 110) of the online survey on pain assessment and management in pet rabbits.

	Drug	% (*n*)	Dosage range in mg/kg (number of provided answers)	Median (IQR)	Route of administration (number of provided answers)
NSAIDs	Meloxicam	63.6 (70)	0.06‒5.00 (60)	0.50 (0.50)	SC (37), IM (1), IV (1), PO (4)
	Carprofen	‒	‒	‒	‒
Pyrazolone	Metamizole	60.0 (66)	20.0‒75.0 (62)	50.0 (3.0)	SC (33), IM (4), IV (8), PO (3)
Opioids	Buprenorphine	13.6 (15)	0.03‒50.00 (10)	0.03 (0.02)	SC (4), IM (3), IV (1)
	Butorphanol	17.3 (19)	0.02‒3.00 (14)	0.60 (0.73)	SC (2), IM (9), IV (2)
	Fentanyl	5.5 (6)	0.01‒0.02 (4)	0.02 (0.01)	IM (2), IV (1)
	Tramadol	0.9 (1)	‒	‒	‒
Local anaesthetics	Lidocaine	10.0 (11)	0.5‒50.0 (7)	2.0 (3.0)	L (6), SC (1)
	Bupivacaine	‒	‒	‒	‒
Anaesthetics with analgesic effect	Ketamine	29.1 (32)	0.2‒50.0 (22)	20.0 (12.0)	SC (1), IM (11), IV (1)
	Dexmedetomidine	9.1 (10)	0.03‒0.25 (7)	0.10 (0.20)	IM (4)
	Medetomidine	25.5 (28)	0.1‒25.0 (19)	0.2 (0.2)	SC (2), IM (12)
	Xylazine	3.6 (4)	2.0 + 4.0 (2)	‒	IM (1)

*Note*: Please be aware that some of the dosages stated by survey participants are outside of the dosage range found in literature and can be harmful for the animals. Respondents were able to select and combine multiple drugs. Information on dosages and routes of administration was not provided by all respondents.

Abbreviations: IM, intramuscular; IQR, interquartile range; IV, intravenous; L, local; PO, per os; SC, subcutaneous.

**TABLE 3:** Intraoperative use of analgesics in ovariohysterectomy of rabbits as stated by survey participants (*n* = 110) of the online survey on pain assessment and management in pet rabbits.

	Drug	% (*n*)	Dosage range in mg/kg (number of provided answers)	Median (IQR)	Route of administration (number of provided answers)
NSAIDs	Meloxicam	19.1 (21)	0.2‒1.5 (12)	0.5 (0.6)	SC (8), IV (1)
	Carprofen	‒	‒	‒	‒
Pyrazolone	Metamizole	20.0 (22)	40.0‒65.0 (14)	50.0 (6.3)	SC (6), IM (2), IV (2)
Opioids	Buprenorphine	7.3 (8)	0.03 (3)	‐	SC (2), IM (1), IV (3)
	Butorphanol	7.3 (8)	0.3‒1.0 (5)	0.5 (0.6)	SC (1), IM (3)
	Fentanyl	21.8 (24)	0.004–0.025 (14)	0.020 (0.020)	IM (9), IV (8), CRI (5)
	Tramadol	‒	‒	‒	‒
Local anaesthetics	Lidocaine	20.9 (23)	0.1‒4.0 (11)	3.0 (2.5)	L (15), SC (7)
	Bupivacaine	0.9 (1)	1.0‒2.0 (1)	‒	L (1)
Anaesthetics with analgesic effect	Dexmedetomidine	6.4 (7)	0.05‒0.15 (4)	0.09 (0.08)	IM (3), IV (1)
	Medetomidine	32.7 (36)	0.10‒0.33 (21)	0.20 (0)	SC (3), IM (16), IV (3)
	Xylazine	2.7 (3)	‒	‒	SC (1), IM (1)
	Ketamine	40.0 (44)	0.15‒35.00 (23)	12.50 (22.50)	SC (5), IM (16), IV (5), CRI (4)

*Note*: Please be aware that some of the dosages stated by survey participants are outside of the dosage range found in literature and can be harmful for the animals. Respondents were able to select and combine multiple drugs. Information on dosages and routes of administration was not provided by all respondents.

Abbreviations: CRI, constant rate infusion; IM, intramuscular; IQR, interquartile range; IV, intravenous; L, local; SC, subcutaneous.

**TABLE 4:** Postoperative use of analgesics in ovariohysterectomy of rabbits as stated by survey participants (*n* = 110) of the online survey on pain assessment and management in pet rabbits.

	Drug	% (*n*)	Dosage range in mg/kg (number of provided answers)	Median (IQR)	Route of administration (number of provided answers)
NSAIDs	Meloxicam	88.2 (97)	0.1‒2.0 (75)	0.5 (0.5)	SC (16), PO (49)
	Carprofen	0.9 (1)	10.0‒25.0 (1)	‒	SC (1)
Pyrazolone	Metamizole	62.7 (69)	0.04‒75.00 (56)	50.00 (4.50)	IV (2), SC (15), IM (1), PO (34)
Opioids	Buprenorphine	10.0 (11)	0.03‒0.20 (7)	0.03 (0)	IV (2), SC (4), IM (1)
	Butorphanol	0.9 (1)	0.02 (1)	‒	SC (1)
	Fentanyl	0.9 (1)	0.02 (1)	‒	‒
	Tramadol	3.6 (4)	10.0‒50.0 (4)	11.3 (30.6)	PO (2)
Local anaesthetics	Lidocaine	‒	‒	‒	‒
	Bupivacaine	‒	‒	‒	‒
Anaesthetic drugs with analgesic effect	Ketamine	4.5 (5)	0.15‒20.00 (3)	0.20 (19.85)	IM (2)
	Dexmedetomidine	1.8 (2)	0.25 (1)	‒	IM (1)
	Medetomidine	3.6 (4)	0.20‒0.33 (3)	0.25 (0.13)	IM (1)
	Xylazine	0.9 (1)	‒	‒	‒

*Note*: Please be aware that some of the dosages stated by survey participants are outside of the dosage range found in literature and can be harmful for the animals. Respondents were able to select and combine multiple drugs. Information on dosages and routes of administration was not provided by all respondents.

Abbreviations: IM, intramuscular; IQR, interquartile range; IV, intravenous; PO, per os; SC, subcutaneous.

Preoperative analgesia was provided by 75.5% (83/110) of veterinarians, whereas 24.5% did not administer preoperative analgesia. Meloxicam (63.6%, 70/110) and metamizole (60.0%, 66/110) were the most frequently mentioned analgesics in this regard. More than half of the respondents (54.5%, 60/110) stated that they combined meloxicam and metamizole preoperatively. Opioids, mainly butorphanol (17.3%, 19/110) and buprenorphine (13.6%, 15/110), were used by 34.5% (38/110) of veterinarians in the preoperative phase. A total of 28.2% (31/110) stated that they combined opioids with meloxicam, metamizole or both; 10.0% (11/110) stated that they used lidocaine preoperatively, administering it either locally, for incisional infiltration (6/110), subcutaneously (1/110) or not further specified (4/110).

#### Intraoperative analgesia

A total of 31.8% (35/110) of veterinary surgeons gave opioids intraoperatively; 21.8% (24/110) used fentanyl, 7.3% (8/110) used buprenorphine and the same number used butorphanol. Meloxicam, metamizole or both were administered intraoperatively by 26.4% (29/110). A total of 21.8% (24/110) used local anaesthetics (23/110 lidocaine and 1/110 bupivacaine). Most of these participants stated that they applied lidocaine on the ligamentum latum of the ovaries or ‘in the operating field’ (15/110). Seven participants used lidocaine for wound infiltration.

Concerning anaesthetics with analgesic effect, 29.1% (32/110) reported using ketamine preoperatively and almost all of these participants (31/110) combined ketamine with an alpha‐2 agonist. A total of 40.0% (44/110) used ketamine intraoperatively. The most frequently used alpha‐2 agonist intraoperatively was medetomidine (32.7%, 36/110).

Five respondents stated that they used no drugs with analgesic effect pre‐ or intraoperatively but only in the postoperative phase.

#### Postoperative analgesia

A total of 95.5% (105/110) of veterinarians stated that they used analgesics in the postoperative phase, and 87.3% (96/110) used them for more than 24 hours and up to 7 days postoperatively.

A total of 88.2% (97/110) administered meloxicam and 62.7% (69/110) used metamizole postoperatively. A total of 58.2% (64/110) combined both drugs postoperatively. Next to the reported dosages, administration intervals and number of treatment days were highly variable, especially for metamizole (Table 5). Only 14.5% (16/110) of participants reported using opioids postoperatively, with buprenorphine being the most common (10.0%, 11/110). None of the participants stated that they used local anaesthetics post‐surgery.

**TABLE 5:** Reported dosing intervals and treatment days for the three most commonly used postoperative analgesics in rabbits undergoing ovariohysterectomy as stated by survey participants.

Drug	Dosing interval (number of provided answers)	Treatment days, categorised (number of provided answers)
Meloxicam	q24h (17), q24‐q12h (8), q12h (9)	<4 (6), 4‒7 (21), 5‒10 (4)
Metamizole	q24h (1), q12h‐q8h (3), q12h‐q6h (3), q8h (2), q8h (6), q8h‐q6h (5), q6h (6), q6h‐q4h (3), q4h (1)	1‒3 (14), 3‒7 (10), 3‒10 (2)
Buprenorphine	q24h‐q12h (1), q12h (1), q8h‐q6h (1)	1 (3), ≥1 (1)

*Note*: q24h = every day, q12h = every 12 hours, q8h = every 8 hours, q6h = every 6 hours, q4h = every 4 hours. Information on dosing intervals and treatment days was not provided by all respondents.

According to 81.8% (90/110) of the participants, rabbits were discharged on the day of surgery. A total of 13.6% (15/110) discharged patients on the day after surgery, and only 4.6% (5/110) stated that they generally hospitalised rabbits for more than 24 hours post‐surgery. Almost all veterinarians (90.0%, 99/110) reported conducting a follow‐up check on rabbits after performing an OHE.

#### Use of multimodal analgesia and reported satisfaction with analgesic therapy

According to the definition of multimodal analgesia as the use of pharmacological agents targeting different nociceptive receptors and pathways to induce analgesia,^27^ all but five participants in our study provided multimodal analgesia in the pre‐ and intraoperative phases combined. The proportion in the postoperative phase was 60.0% (66/110), of which 78.8% (52/66) were combinations of metamizole and meloxicam only. Overall, 60.0% (66/110) administered both NSAIDs and opioids perioperatively. Participants who graduated after 2010 were more likely to use opioids perioperatively (72.7% vs. 53.0%), with a statistically significant association (*p* = 0.047).

Overall, 84.5% (93/110) of veterinarians operating OHEs felt that rabbits received sufficient analgesia during and after surgery.

## DISCUSSION

The present study provides information on pain recognition and pain management in rabbits with a focus on analgesic therapy in ovariohysterectomies as reported by German‐speaking veterinarians.

The majority of veterinarians (83.8%) felt fairly or even very confident in identifying pain in rabbits. A comparable result was found in the survey of Benato et al.,^25^ in which 70% of respondents stated that they felt either ‘fairly confident’ or ‘very confident’ in recognising signs of pain in rabbits. In the very first survey study on analgesic therapy in rabbits, conducted by Keown et al.^24^ in New Zealand, 76.9% rated their knowledge of pain recognition in rodents and rabbits as inadequate. The participants in the aforementioned paper reported that rabbits and rodents accounted for no more than 15.0% of their total caseload, which may have left them less experienced in treating rabbits compared to the participants in this survey. Furthermore, numerous publications on recognising and treating pain in rabbits have been published over the last decade, potentially increasing veterinarians' confidence in the subject.

In accordance with Benato et al.,^25^ only a minority of veterinarians stated that they used the RbtGS to assess pain, but with 23.4% compared to 12% the amount in this study was slightly higher, and another 26.6% stated that they analysed facial expressions but did not specifically mention the use of the RbtGS. The fact that participants in this survey who had graduated later than 2010 used the RbtGS more frequently could indicate that information on pain scaling was either more readily available to them and/or that there was an increased interest in the subject. No participant mentioned the use of CANCRS, a composite pain scale for rabbits from 2020 that combines the RbtGS with clinical parameters,^7^ or the BRPS, a multidimensional pain scaling method for rabbits that was published a year before the survey was conducted.^5^ There was also no mention of the RPBS, a behavioural scale developed for assessing postoperative pain in rabbits that was published only 2 months before the survey began.^6^ Nevertheless, the results show that some participants combined aspects assessed in complex pain scales, such as the BRPS, by analysing posture alongside facial expressions and behaviour. In retrospect, it might have been advantageous to ask specifically about the use of developed pain‐scaling methods for rabbits in a separate question, or to further specify the question about indicators of pain in this regard. However, an open‐ended question was chosen in order to gather unprompted answers.

It is noticeable that a vast majority of respondents felt that their time at university had prepared them inadequately or not at all for the treatment of pain in rabbits and small mammals. It seems that a large proportion of the surveyed veterinarians had gained knowledge via further education and routinely working with rabbits after graduating. This is evident from the high level of confidence in pain assessment and the high level of satisfaction with the chosen pain therapy.

Veterinarians reported using a wide range of analgesic drugs in OHEs in rabbits. Similar to the findings of Benato et al.,^25^ dosage ranges for all drugs varied widely, which could be due to the still insufficient number of studies on analgesic therapy in rabbits and the associated lack of standards in pain therapy for the species. It may also be an indication that participants had no or only partial access to existing study results. It should also be pointed out that the participants' answers on dosages of analgesic medications, specified as mg/kg, had to be typed in manually rather than being selected from a drop‐down menu or similar. This gave the participants the opportunity to provide unbiased answers, but at the same time, this may have resulted in some participants either not reporting their dosages at all or making typing errors when entering them. It is also possible that some of the respondents intended to write down the dosage in mg/kg but ultimately confused it with mL/kg, entered their dosages in the wrong column or similar. This may have led to some dosage ranges being particularly large (e.g., a dosage range of buprenorphine 0.03‒50.00 mg/kg in the preoperative phase or a dosage range of 0.04‒75.00 mg/kg for metamizole in the postoperative phase). It should be pointed out that some of the reported dosages (e.g., 50.00 mg/kg of buprenorphine) can be harmful for the animals if administered. Still, it was decided that all specified dosages should be included in the results section.

While it is positive that three‐quarters of veterinarians use preoperative analgesia when performing OHEs in rabbits, this might still not be ideal, as pre‐emptive analgesia before surgery was found to be valuable in cats and dogs, reducing postoperative pain^28^, ^29^ and is considered a part of a modern, proactive pain management.^30^ However, this amount is comparable to the administration of preoperative analgesia in dogs in one survey study.^31^

Anaesthetics with an analgesic effect were reported by participants in the questions concerning pre‐ and intraoperative as well as postoperative analgesics. Although ‘preoperative’ was defined as a time period up to 2 hours before the surgery, it is possible that respondents interpreted the time point for the actual start of anaesthesia differently and thus stated the anaesthetics they used to induce anaesthesia partly in the pre‐ and intraoperative section. Except for six participants, there was no duplication between pre‐ and intraoperative administration of anaesthetics with analgesic effect. The participants who stated that they used them postoperatively did not comment on their reasons for doing so.

A large proportion of veterinarians in this study seemed to use multimodal pain therapy in rabbits, which is considered vital in modern veterinary pain management.^30^, ^32^, ^33^ In contrast to the present study, Benato et al.^25^ refer to multimodal analgesia as an explicit combination of NSAIDs and opioids only. This was carried out perioperatively by 70% of veterinarians treating rabbits surgically in the same paper. According to this definition, the figure is slightly lower in our study, at 60.0%. In addition, other drugs with an analgesic effect should also be included in the definition of multimodal pain therapy.

Interestingly, lidocaine was used pre‐ and intraoperatively by some participants, yet it was not reported to be used at all postoperatively, despite Schnellbacher et al.^34^ having demonstrated that the postoperative administration of lidocaine constant rate infusion in rabbits, particularly following OHEs, positively impacted feed intake, gastrointestinal motility and faecal output, as well as blood glucose levels and behavioural parameters. Again, it is possible that these results are not yet widely known despite the availability of the referenced paper and numerous others on analgesic therapy in rabbits via open access.

It should also be noted that many veterinarians stated that they used the pyrazolone metamizole in combination with meloxicam. Metamizole has been completely withdrawn from the market in many countries, including the UK, mainly due to its risk of causing agranulocytosis in humans.^35^ The frequent use of this drug in rabbits by participants in this study can be considered as a locally established aspect of pain therapy in Germany. Metamizole was also found to be the most prescribed painkiller in human medicine in Germany in a study from 2019.^36^ Injectable solutions of metamizole are licensed in Germany for use in livestock, horses and dogs, and an oral formulation from human medicine can be used off‐label in rabbits. While the analgesic mechanism of action for metamizole is not fully clarified,^37^ and while there is currently little knowledge on the analgesic effect of metamizole in rabbits,^38^, ^39^ it seems that participants often used the drug with confidence. It should be noted that the reasons behind the selection of a particular analgesic were not provided by the study participants, nor were they explicitly queried.

The majority of veterinarians (87.3%, 96/110) used analgesics for more than 24 hours after surgery, and up to 7 days after an OHE, and thus often discharged the patients with analgesics, as was the case in the paper of Benato et al.^25^ One study from the UK in cats and dogs reported smaller numbers, with 75.1% of dogs and 33.4% of cats receiving analgesics following discharge after routine surgery.^40^ In an Australian study, only 24% of dogs received ongoing analgesia after discharge following an OHE.^31^ As both studies were conducted about a decade ago, and analgesic protocols have certainly changed since then, these results should be compared with those of this study with caution.

As five participants stated that they did not use analgesic drugs until after surgery, it is unclear whether this information was provided incorrectly or whether the questions were misunderstood. The same applies to the five other cases in which veterinarians stated that they did not use analgesics postoperatively. All of these practices would be in clear violation of ethical consensus on animal welfare.

Non‐pharmacological options of pain management were not specifically addressed in this survey, and participants made no comments on the use of alternative medicine. Several participants mentioned that their aim was to discharge the rabbits into their familiar environment as early as possible, which might contribute to a fast recovery and improved wellbeing of their patients.

There are several limitations to this study. Various veterinarians were initially addressed with the invitation to participate in the survey, but it is expected that those with a particular interest in small mammal medicine were more likely to participate than others. The respondents could only fill out the survey form online, which may have caused participants to abandon the survey before completing it due to technical difficulties. Some people might also be more inclined to participate in an offline survey. In addition to the relatively small sample size, there is an evident selection bias in the survey responses, with 90.3% of participants being female. This is significantly higher than the 70% proportion of female practising veterinarians in Germany.^41^ This should be taken into account, since several studies showed significant effects of gender on pain perception and analgesic treatment in veterinary medicine, with female veterinarians giving higher pain scores and perceiving certain procedures as more painful than male veterinarians.^14^, ^15^, ^22^, ^23^, ^42^, ^43^ It is possible that more female veterinarians are interested in small mammal medicine in Germany and were therefore more motivated to partake in the survey. Veterinarians with a certified specialisation in small mammal medicine in Germany are mainly female.^44^ There was also a high percentage of female respondents (77.6%) in the only other survey study on pain assessment and management in rabbits carried out in the last decade (in the UK).^25^

In conclusion, the results of this survey reveal clear differences among German‐speaking veterinarians regarding pain assessment and the analgesic treatment of rabbits during OHEs. The subjective confidence of the participants in the assessment of pain and their satisfaction with their chosen pain therapy were high overall. Respondents used a variety of indicators and often combined them to detect pain in rabbits. The choice of analgesic drugs and dosages varied widely. There was a lack of pre‐emptive analgesia in a quarter of the treatment plans provided. The use of opioids, especially in the postoperative period, was low, but the overall perioperative use of opioids in combination with NSAIDs was comparable to the findings of Benato et al.^25^ The frequent use of metamizole by respondents in this study was an important difference which stood out.

The vast majority of participants stated that they had been prepared inadequately or even not at all by their university on how to treat pain in rabbits. This deficit in German veterinary education should be addressed. An improvement in pain management in rabbits could also be achieved by increasing veterinarians’ interest and providing a wider range of continuing education and training on the subject. Further studies, especially on multimodal analgesia in rabbits and on changes in analgesic therapy over time, are needed.

## AUTHOR CONTRIBUTIONS

*Conceptualisation, data curation, investigation, formal analysis, methodology and writing—original draft preparation, review and editing*: Stephanie Zein.

*Formal analysis, statistical consulting, methodology and writing—review and editing*: Katharina C. Jensen.

*Conceptualisation, methodology, project administration, supervision and writing—review and editing*: Kerstin Müller.

## CONFLICT OF INTEREST STATEMENT

The authors declare no conflicts of interest.

## ETHICS STATEMENT

The authors confirm that the ethical policies of the journal, as noted on the journal's author guidelines page, have been adhered to. No further ethical approval was required.

## Supporting information



Supporting Information

## Data Availability

The data that support the findings of this study are available from the corresponding author upon reasonable request.
