# Supplementary material for: Survey of German veterinarians' approaches to pain assessment and management of perioperative pain in pet rabbits
Source: Vet Rec. 2025 Dec 4;198(3):e92–e100. doi: 10.1002/vetr.6018 (PMC12857525; doi:10.1002/vetr.6018)
Supplement: Supplementary file 1 — Supporting Information [file VETR-198--s001.docx]

## **Appendix**

English translation of the German questionnaire:

Online survey on pain assessment and management in pet rabbits

**1)** How often do you treat rabbits in your practice or clinic? (single-response multiple-choice question)

- Less than 1 time per month
- 1-3 times per month
- Weekly
- Multiple times per week
- Daily
- Other:

**2)** How confident do you feel in recognising and assessing pain in rabbits? (single-response multiple-choice question)

- Insecure
- Rather insecure
- Fairly confident
- Very confident
- Other:

**3)** How do you generally assess pain in rabbits? What indicators do you use? (free-text answer)

**4)** How did your university education prepare you to treat pain in rabbits and small mammals in general? (single-response multiple-choice question)

- Very well
- Well
- Sufficiently
- Inadequately
- Not at all

**5)** Do you perform ovariohysterectomies (OHEs) in rabbits? (single-response multiple-choice question)

- Yes
- No

**6)** How often do you perform ovariohysterectomies (OHEs) in rabbits? (single-response multiple-choice question) (*if respondents answered question no. 5 with “yes”)*

- 1-3 times per month
- Weekly
- Daily
- Other:

**7)** When is a rabbit typically brought into your practice / clinic for a planned ovariohysterectomy (OHE)? (single-response multiple-choice question) *(if respondents answered question no. 5 with “yes”)*

- On the day of surgery
- The day before surgery
- Other:

**8)** Do you administer analgesics to a rabbit undergoing an ovariohysterectomy (OHE) preoperatively (within 2 hours before surgery)? (single-response multiple-choice question)

- Yes
- No

**9) Please select below which analgesics you routinely use for an ovariohysterectomy (OHE) in rabbits. (Prerequisite: The animal has no pre-existing conditions. It is a juvenile animal, approximately one year of age, ASA classification Group 1).**

What analgesics do you use preoperatively (within 2 hours before surgery) for a rabbit undergoing an ovariohysterectomy (OHE)? *(if respondents answered question no. 5, 8 with “yes”)*

(multiple-response question, please enter your standard dosage used in mg/kg and route of drug administration directly behind the selected drug)

- Ketamine (mg/kg):
- Dexmedetomidine (mg/kg):
- Medetomidine (mg/kg):
- Xylazine (mg/kg):
- Metamizole (mg/kg):
- Meloxicam (mg/kg):
- Buprenorphine (mg/kg):
- Butorphanol (mg/kg):
- Fentanyl (mg/kg):
- Tramadol (mg/kg):
- Bupivacain (mg/kg):
- Mepivacaine (mg/kg):
- Lidocaine (mg/kg):
- Gabapentin (mg/kg):
- Other:

**10)** Do you administer analgesics to a rabbit undergoing an ovariohysterectomy (OHE) intraoperatively (immediately during the surgical procedure)? (single-response multiple-choice question)

- Yes
- No

**11)** What analgesics do you use intraoperatively (during surgery) for a rabbit undergoing an ovariohysterectomy (OHE)? *(if respondents answered question no. 5, 10 with “yes”)*

(multiple-response question, please enter your standard dosage used in mg/kg and route of drug administration directly behind the selected drug)

- Ketamine (mg/kg):
- Dexmedetomidine (mg/kg):
- Medetomidine (mg/kg):
- Xylazine (mg/kg):
- Metamizole (mg/kg):
- Meloxicam (mg/kg):
- Buprenorphine (mg/kg):
- Butorphanol (mg/kg):
- Fentanyl (mg/kg):
- Tramadol (mg/kg):
- Bupivacain (mg/kg):
- Mepivacaine (mg/kg):
- Lidocaine (mg/kg):
- Gabapentin (mg/kg):
- Other:

**12)** Do you administer analgesics to a rabbit undergoing an ovariohysterectomy (OHE) postoperatively (within a period of up to seven days post-op)? (single-response multiple-choice question)

- Yes
- No

**13)** For how long do you administer analgesics to a rabbit after an ovariohysterectomy (OHE)? (multiple-response multiple-choice question) *(if respondents answered question no. 5, 12 with “yes”)*

- On the day of surgery
- For more than 24hrs and up to seven days post-surgery
- Other:

**14)** What analgesics do you use postoperatively (after surgery) for a rabbit undergoing an ovariohysterectomy (OHE)? *(if respondents answered question no. 12 with “yes”)*

(multiple-response question, please enter your standard dosage used in mg/kg and route of drug administration as well as duration of treatment directly behind the selected drug)

- Ketamine (mg/kg):
- Dexmedetomidine (mg/kg):
- Medetomidine (mg/kg):
- Xylazine (mg/kg):
- Metamizole (mg/kg):
- Meloxicam (mg/kg):
- Buprenorphine (mg/kg):
- Butorphanol (mg/kg):
- Fentanyl (mg/kg):
- Tramadol (mg/kg):
- Bupivacain (mg/kg):
- Mepivacaine (mg/kg):
- Lidocaine (mg/kg):
- Gabapentin (mg/kg):
- Other:

**15)** When do you typically discharge a rabbit after an ovariohysterectomy (OHE)? (single-response multiple-choice question) (*if respondents answered question no. 5 with “yes”)*

- On the day of surgery
- The day after surgery
- After a hospital stay of more than 24 hours
- Other:

**16)** Do you see rabbits after an ovariohysterectomy (OHE) for a follow-up examination, and if so, when? (single-response multiple-choice question) (*if respondents answered question no. 5 with “yes”)*

- Yes
- No
- Other:

**17)** Do you feel that the rabbits are receiving adequate analgesia during and after your ovariohysterectomy (OHE) procedures? (single-response multiple-choice question) (*if respondents answered question no. 5 with “yes”)*

- Yes
- No
- Other:

**18)** What is your gender? (single-response multiple-choice question)

- Female
- Male

**19)**  In which country do you work? (single-response multiple-choice question)

- Germany
- Austria
- Switzerland
- Other:

**20)** Which federal state do you live in? *(if respondents answered question No. 19 with “Germany”*) (single-response multiple-choice question)

- Baden-Württemberg
- Bavaria
- Berlin
- Brandenburg
- Bremen
- Hamburg
- Hessen
- Mecklenburg-Vorpommern
- Lower Saxony
- North Rhine-Westphalia
- Rhineland-Palatinate
- Saarland
- Saxony
- Saxony-Anhalt
- Schleswig-Holstein
- Thuringia

**21)** In what year did you obtain your veterinary license? (drop-down menu of years to select)

**22)** Since when have you been practicing as a veterinarian? (drop-down menu of years to select)

**23)** Are you specialised in treating small mammals, including rabbits, as a certified specialist or are you listed on the DVG-AG Kleinsäuger list? (multiple-response multiple-choice question)

- Yes, I am a certified specialist in small mammal medicine
- Yes, I have an additional qualification in small mammal medicine
- Yes, I am listed on the DVG-AG Kleinsäuger list
- None of the above, but I regularly attend continuing education courses in small mammal medicine (minimum two per year)
- None of the above

**24)** Do you work in a practice or clinic with a specialist or department for small mammal medicine? (single-response multiple-choice question)

- Yes
- No
